# Coupling a simple irradiance description to a mechanistic growth model to predict algal production in industrial-scale solar-powered photobioreactors

**DOI:** 10.1007/s10811-016-0892-6

**Published:** 2016-06-21

**Authors:** Philip Kenny, Kevin J. Flynn

**Affiliations:** College of Science, Swansea University, Swansea, SA2 8PP UK

**Keywords:** Microalgae, Biomass, Biofuels, Modelling, Photobioreactor

## Abstract

Various innovative photobioreactor designs have been proposed to increase production of algae-derived biomass. Computer models are often employed to test these designs prior to construction. In the drive to optimise conversion of light energy to biomass, efforts to model the profile of irradiance levels within a microalgal culture can lead to highly complex descriptions which are computationally demanding. However, there is a risk that this effort is wasted if such optic models are coupled to overly simplified descriptions of algal physiology. Here we demonstrate that a suitable description of microalgal physiology is of primary significance for modelling algal production in photobioreactors. For the first time, we combine a new and computationally inexpensive model of irradiance to a mechanistic description of algal growth and test its applicability to modelling biofuel production in an advanced photobioreactor system. We confirm the adequacy of our approach by comparing the predictions of the model against published experimental data collected over a 2 ½-year period and demonstrate the effectiveness of the mechanistic model in predicting long-term production rates of bulk biomass and biofuel feedstock components at a commercially relevant scale. Our results suggest that much of the detail captured in more complicated irradiance models is indeed wasted as the critical limiting procedure is the physiological description of the conversion of light energy to biomass.

**Electronic supplementary material:**

The online version of this article (doi:10.1007/s10811-016-0892-6) contains supplementary material, which is available to authorized users.

## Introduction

In the drive to increase production of algae-derived biomass for industrial application, various innovative photobioreactor designs have been proposed. Such novelty is often manifested in the deployment of intricate geometries to optimise light availability throughout the culture suspension by balancing the volume to surface area ratio while at the same time addressing issues related to fluid dynamics and aeration (Grobbelaar 2009; Posten 2009; Wang et al. 2012). Other works emphasise factors such as the direct light incidence angle, the refractive index of the medium, photon scattering in dense cultures and levels of diffuse irradiance (Molina Grima et al. 1999; Pilon et al. 2011; Slegers et al. 2011, 2013).

Models describing light availability for photosynthesis in algal suspensions have common features: some means to describe the level of light incident to the culture surface and a measure of the extent to which that light is attenuated by the medium (including the growing algal suspension) through which it passes. Surface irradiance may, as appropriate, be input as a fixed parameter or calculated functionally. The latter can range from a simple step function description of artificial light being switched on and off to equations linked to the annual and diel solar cycles (Walsby 1997) and applied according to the choice of location and setup of the system being studied. Light attenuation is most commonly modelled according to a Beer-Lambert description, and for most purposes, it provides an adequate approximation (Lee 1999) despite the fact it can either over- or under-estimate the available light depending on whether scattering or absorption effects are more dominant. It is difficult to separately quantify the effects of scattering vs absorption experimentally (Acién Fernández et al. 1997), with the impact on the organisms depending on the action spectrum of the photosystems as the cells acclimate to changes in light quantity and quality and to changes in their nutrient status. From a purely pragmatic perspective, there are the challenges of measuring the light profile inside dense algal suspensions within narrow optic paths in units that are meaningful for the organisms (i.e. photosynthetically active photon flux density, taking into account the spectrum of light absorbance by algal pigments). It is far more straightforward to measure the net light attenuation and to consider it as a function of depth (i.e. distance from the illuminated surface), cell concentration and pigment content, as has been done for oceanographic applications (Fasham et al. 2006).

Determining full solutions to the radiative transfer equation offers a more robust approach than relying on Beer-Lambert approximations (Pilon et al. 2011), but is a far more computationally intense task (Cornet et al. 2003). As a compromise, it is possible to make simplifications (possibly combined with empirical measurements (Krujatz et al. 2015)) and still, if so desired, be able to calculate the full irradiance profile within the bioreactor (Cornet and Dussap 2009).

Whatever the level of sophistication, there is a danger that the value of such attention to detail for modelling optics can be diminished (de facto wasted) if the model describing algal growth and physiology, to which the irradiance model is coupled, is too simple. Such simplifications may be justified for descriptions of steady-state or light-limited conditions that are only able to capture certain aspects of growth dynamics (Takache et al. 2010, 2012). However, there are clear dangers in deploying simple biological descriptions under non-steady-state situations. For instance, merely relating productivity projections to a fixed estimated photosynthesis efficiency factor can lead to predictions that are a whole order of magnitude greater than are actually achievable using even the most advanced and finely optimised growth systems currently in operation (Weyer et al. 2010; Wigmosta et al. 2011). Problems become apparent in simple Monod-type descriptions of light-photosynthesis interactions (Béchet et al. 2013), where the maximum photosynthesis rate and half-saturation intensity are cell concentration dependent (Jeon et al. 2005) which, in turn, is itself a (sometimes highly) dynamic variable dependent on the rate of photosynthesis. Coupling dynamic variables with a steady-state model is akin to putting a round peg in a square hole. Unless the means to fully capture the physiological dynamics correctly are in place, especially with respect to the changing balance between light and nutrient limitation (Flynn 2003a), the coupled abiotic-biotic model as a whole can become dysfunctional (Flynn 2003b). This matter is of particular importance because modulating co-light/nutrient limitation is a crucial factor affecting microalgal production for different feedstocks (Kenny and Flynn 2015).

Photoacclimation is an important (although, in the context of industrial production, often neglected) physiological process that affects not only the description of irradiance in the algal suspension but also the conversion of light energy into biomass. In this process, the phototrophic microalgae increase their photopigment content to raise energy capture from a diminishing light field. This becomes a self-propagating process as the collective cell population increase in pigment content in the individual cells decreases light penetration further. In contrast, with nutrient exhaustion, and/or with an increase in irradiance received by the cells, pigment is lost to mitigate against the procurement of excess photoreductant; under such conditions, photoacclimation decreases the absorbance of light by the algal suspension. Photoacclimation is most simply described by the algal chlorophyll to C-biomass ratio (Chl:C), ranging to a maximum mass ratio of ca. 0.06. The process displays a diel cycle in Chl:C during a light-dark cycle. For further information and for modelling approaches, see Flynn et al. (2001) and Flynn (2003a).

The time scale over which all these dynamic abiotic and biotic changes occur also has a bearing on whether a given combination of irradiance and physiological model is appropriate or not. Under natural light, the vagaries of cloud cover, the sun’s position relative to surrounding obstructions and the like can produce sudden changes to the surface irradiance, while an individual cell within a turbulent environment will also be exposed to rapidly varying levels of light due to its position relative to the illuminated surface (Molina Grima et al. 1999; Posten 2009). These changes can occur in the order of milliseconds to seconds, and capturing the associated fluid dynamics computationally may require a time step of the order of microseconds to maintain numerical stability (Lobatón et al. 2011; Seo et al. 2012). By contrast, the computational time step required to capture physiological events in microalgae in the physiological model may be the equivalent of several simulation minutes (for simpler descriptions) and even hours (Flynn 2003b). There is thus a potential mismatch in time scale between physics and biology sub-models of up to 8 orders of magnitude. Hence, the rate of flow within the system (Molina et al. 2001; Li et al. 2015) can be such that several panels’ worth of culture volume may have passed through a given point between two time steps for biology models and any one of these transient volume elements is equivalent to another. Thus, in the context of the biological model (and for the experience of the average algal cell), it seems appropriate to consider a well-mixed culture as, indeed, being homogeneous, which allows us to integrate over the panel depth to calculate the average photosynthetic activity in the water column in the same way as one would for a natural mixed water column of many metres depth (Fasham et al. 2006). By extension, such homogeneity (which in bioreactors would apply to the equivalent of mixed layer depths of only a fraction of 1 m, if not only a few cm) should make it possible to average the surface irradiance over the whole illuminated area.

In this work, we eschew much of the complexity that might be associated with irradiance models to consider production in an outdoor pilot-scale flat panel reactor using a computationally inexpensive description of the average photon flux density (PFD) hitting a panel surface and attenuated within the medium according to a Beer-Lambert description. We confirm the adequacy of this approach by comparing the predictions of a series of biological model descriptions of varying sophistication against experimental data. Furthermore, we also demonstrate the effectiveness of an acclimative, mechanistic model of algal growth to predict large-scale, long-term production rates of bulk biomass and biofuel feedstock components in such a system.

## Methods

### Modelling irradiance and photosynthesis

Bioreactors may be lit artificially or naturally. In the former, the surface irradiance *E*_0_ driving photosynthesis is under tight control. In an outdoor reactor, *E*_0_ depends upon geographical location, time of year and atmospheric conditions. In what follows, we describe *E*_0_ for a naturally lit reactor.

Latitude informs a solar cycle function which simulates diurnal and seasonal variations in available natural light:

1 $$ {E}_0=\mathrm{S}\mathrm{C}\left( \sin \varphi \sin \delta - \cos \varphi \cos \delta \cos \theta \right)\varLambda $$

where SC is the solar constant, *φ* is the latitude, *δ* is the solar declination angle and *θ* is the angular description of the diel solar cycle. This expression is multiplied by an average insolation clearness index *Λ* (typically, between 0.45 and 0.8) for each latitude and month obtained from NASA’s Surface Meteorology and Solar Energy database (eosweb 2016). This clearness index defines the fraction of sunlight penetrating the atmosphere on an average day (accounting for cloud cover, dust, etc.).

**Fig. 1:**
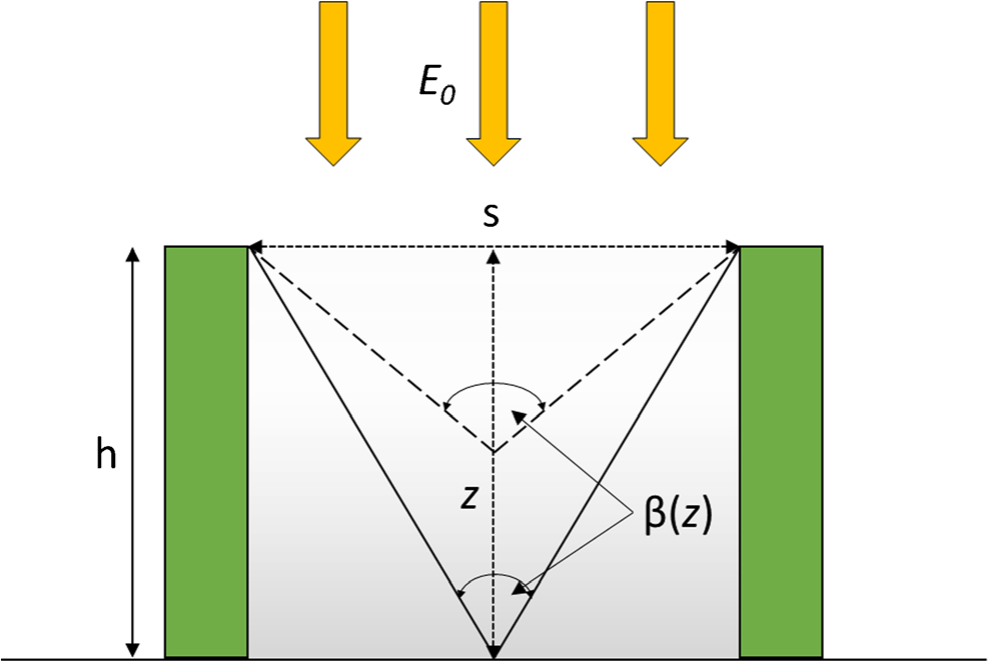
A typical irradiance profile between two vertical panels (*flanking rectangles*) of height *h*, separated by distance *s*. The incident irradiance at any height *z* on the surface of each panel is a function of the current solar irradiance *E*_0_ and the angle formed between the diagonals, *β*(*z*) (see Eq. 2). The average irradiance is calculated across the whole reactor panel surface (see Eq. 3). Additional irradiance (such as through any neighbouring panels) penetrating the dense algal suspension inside the panels is neglected

One factor that is often neglected in irradiance descriptions for outdoor reactors is that the irradiance calculated in Eq. 1 is the vertical component of the incoming sunlight vector and hence is a vector quantity itself, but one which is normal to the Earth’s surface. The resulting light energy vector field per unit area can be measured in terms of the PFD. Thus, for a typical array of bioreactor panels, we may consider the irradiance as coming from directly overhead (see Fig. 1) and, as a first approximation, that the amount of light at any height at the sides of the vertical panel then depends upon the proportion of sky that is directly visible at that point and unobstructed by the neighbouring panels (i.e. we assume the culture is dense enough to neglect any attenuated light penetrating the neighbouring panels). For an industrial-scale reactor where the panel length is much greater than the panel separation, we can neglect end effects and use cylindrical symmetry to define the angle subtended by the unobstructed sky (from one horizon to the opposite) as being *π* radians, while the angle subtended by an obstructed view between any two panel is the angle *β* (see Fig. 1). Using elementary geometry, *β* is a function of the height *z* from a point level with the top of the panel and the panel separation *s* according to *β* = 2 tan^− 1^(*s*/2*z*). Thus, at any *z* between the top of a panel of height *h* (*z* = 0) and the bottom (*z = h*), the irradiance hitting the surface as a function of height is given by

2 $$ E(z) = \frac{E_0\beta (z)}{\pi }=\frac{2{E}_0}{\pi }{ \tan}^{-1}\left(\frac{s}{2z}\right) $$

To calculate the average surface irradiance $\overline{E_0}$ over the whole panel side, we integrate Eq. 2 from *z =* 0 to *z = h* and divide by the height of the panel:

3 $$ \overline{E_0}\ \begin{array}{l}={\displaystyle \underset{0}{\overset{h}{\int }}}\frac{E(z)}{h}\ \mathrm{d}z = \frac{2{E}_0}{\pi h}\ {\displaystyle \underset{0}{\overset{h}{\int }}}\ { \tan}^{-1}\ \left(\frac{s}{2z}\right)\ \mathrm{d}z\hfill \\ {}=\frac{E_0}{\pi h}\left[\frac{1}{2}s \ln \left(1+{\left(\frac{2h}{s}\right)}^2\right)+2h{ \tan}^{-1}\left(\frac{s}{2h}\right)\right]\hfill \end{array} $$

In the limit, where the number of panels is large (*N*_p_ → ∞), we can neglect end effects associated with two unobstructed panel sides. A further assumption is that the irradiance levels on both sides of the panel are equal, and therefore, the maximum depth *τ*, to which the light is attenuated according to the Beer-Lambert law, equals half the depth orthogonal to the illuminated sides of the vertical panel (i.e. half the panel width).

The adequacy of the functional description of average irradiance in Eq. 3 was tested against nearly 3 years’ worth of experimental data recorded at the Solix BioSystems research and development facility in Fort Collins, CO, USA (Quinn et al. 2012). The reactor system comprises multiple vertical panels of width, height and length measuring 0.05 × 0.28 × 17.3 m and spaced 0.15 m apart (shown by Quinn et al. (2012) in their supplementary material). The legacy light data were averaged to obtain a mean value for each month while the simulation data were averaged to obtain a mean daily value. As the NASA data are averaged over a 22-year period (as opposed to the 2 ½-year period for the duration of the experiments) and collated over a wider geographical area (1° × 1°), in lieu of more specific on-site data, it was decided in this instance to tune the monthly clearness index *Λ* in Eq. 4 so that the modelled PFD fitted the monthly averaged data as closely as possible. This then allowed the local atmospheric clearness during production to be estimated where direct measurement was not made. As the real values would form a subset of the NASA data, the tuned values for *Λ* were tested for consistency with the NASA data sets. The tuned clearness indices in the simulations ranged between 0.55 and 0.8 and were indeed consistent with the corresponding measurements obtained from the NASA data sets (eosweb 2016) for a site at 40° N, 105° W (Fort Collins, USA).

### Modelling cellular physiology

Total photosynthetic activity, PS, in the water column is calculated by integrating the Smith equation (Smith 1936) which incorporates a Beer-Lambert description (Fasham et al. 2006) over the mixing depth (assuming a homogeneous cell suspension) to obtain

4 $$ \mathrm{P}\mathrm{S} = \frac{\mathrm{Pqm}}{k\tau}\left[ \ln \left(\frac{\overline{E_0}\alpha \mathrm{ChlC}}{\mathrm{Pqm}}+\sqrt{1+{\left(\frac{\overline{E_0}\alpha \mathrm{ChlC}}{\mathrm{Pqm}}\right)}^2}\right)- \ln \left(\frac{\overline{E_0}\alpha \mathrm{ChlC}}{\mathrm{Pqm}}{e}^{-k\tau }+\sqrt{1+{\left(\frac{\overline{E_0}\alpha \mathrm{ChlC}}{\mathrm{Pqm}}{e}^{-k\tau}\right)}^2}\right)\right] $$

where *α* is the photosynthetic efficiency at *E* = 0, ChlC is the mass ratio of chlorophyll to carbon within the microalgae, Pqm is the absolute maximum rate of photosynthesis and *k* is the attenuation factor of the culture. Parameter *k* is a function of the culture medium itself (of minimal impact here but more so if nutrients were to be sourced from digestate that typically contain coloured compounds), and also of the C-specific biomass concentration with its allied pigmentation (ChlC). Pqm, ChlC and *k* are dynamic variables that depend upon the developing biomass and also the nutrient status of the cells, and which must be updated at each time step to capture photoacclimation effects (Flynn et al. 2001). The average photosynthetic activity is thus Eq. 4 divided by the panel depth. (For details of how this is off-set by respiration effects, see Appendix A, in the Supporting material).

**Fig. 2:**
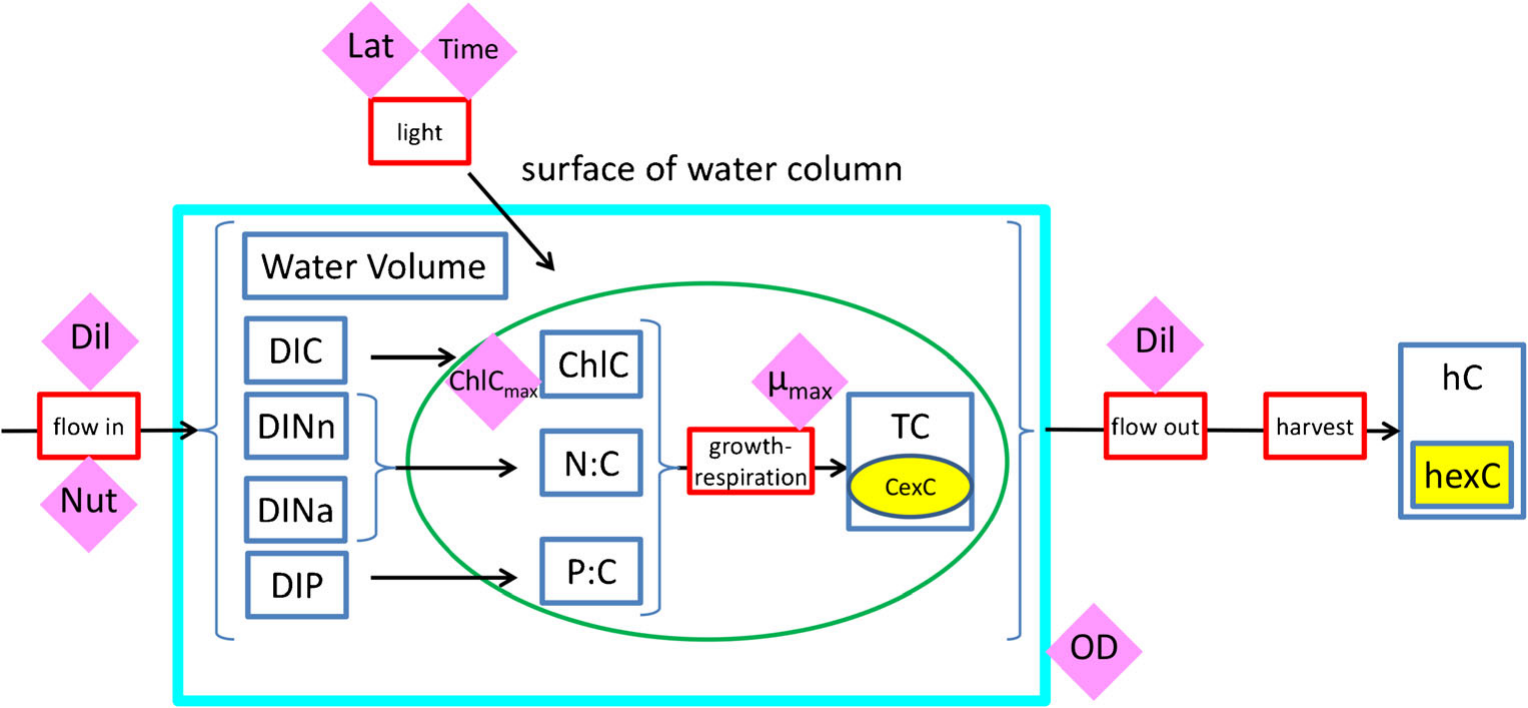
A schematic of the systems biology model structure. Cellular growth and composition are driven by the dynamic interplay of light-limiting and nutrient-limiting factors. Definitions within the model (Flynn 2001) allow for variable cellular C:N:P:Chl stoichiometry and regulated uptake of dissolved nutrients (e.g. DIN, DIP). A proportion (CexC) of biomass is present as C-rich storage products (e.g. fatty acids). See “Methods” for an explanation of state variables within the model, and the online Appendix for equations governing variable C:N:Chl stoichiometry

The fully featured mechanistic model of cellular growth used here is an acclimative construct with varying elemental stoichiometry (Flynn 2001) originally developed for applications to studies of algal ecophysiology and which has been used to investigate issues surrounding the industrial production of bulk biomass and related biofuel feedstocks from microalgae (Flynn et al. 2013; Kenny and Flynn 2015). Appendix B gives details of species and previous scenarios for which this model type has been deployed. As seen in the schematic shown as boxes within Fig. 2, there are a number of state variables defined in the model. Nutrients for algal consumption include dissolved inorganic C (DIC), nitrate (DINn) and/or ammonium (DINa), and phosphate (DIP). The increase in microalgal C-biomass (TC) is a function of the nutrient status of the cells (as cellular N:C and P:C), the status of the photosystems (ChlC) and light. A proportion of biomass is present as C-rich storage products (carbohydrate + lipid; CexC), calculated according to Flynn et al. (2013). Harvested biomass (hC), including such high-C harvested material, is of potential interest for biofuels (hexC).

Constant parameters that are of key concern for controlling productivity, indicated by diamonds in Fig. 2, are as follows: Dil, the dilution rate and harvesting frequency; Nut, nutrient concentration; OD, depth of the culture system (orthogonal to the illuminated surface); Lat, geographic latitude of the facility; Time, day and hour; and *μ*_max_, maximum growth rate of the algae. Lat and Time define the surface irradiance (see Eqs. 1–3), while light available for the microalgae is a function of that surface irradiance over the day-night cycle, absorbance by the algal suspension with reference to OD, TC and ChlC (the latter a function of N:C and of light availability via photoacclimation). The value of hexC depends on TC and CexC, which in turn relates to Dil and Nut, such that growth rate is optimised while N:C is low (noting that N:C is linearly related to N-limited growth potential, when light is non-limiting). Further details regarding functional descriptions of mass balance and cell quota dynamics within this model structure are given by Flynn (2001).

Variations upon this general structure were compared to gauge the level of descriptive sophistication required to adequately capture growth dynamics within a photobioreactor system and accurately predict production rates of bulk biomass and biofuel feedstock components simultaneously. Comparisons were made between the fully acclimative model with its varying C:N:P:Chl stoichiometry, models with varying C:N (but fixed C:P:Chl) and varying C:N:Chl (but fixed C:P) descriptors. An algal model was also deployed featuring fixed stoichiometry, with a C:N:P ratio fixed according to the Redfield ratio (Redfield 1934, 1958). These alternatives are described in Table A.2 in Appendix A. To apply and validate these alternative algal models at a scale relevant to commercial production, we compared each one’s predictions of production against biomass and fatty acid production data from the same study by Quinn et al. (2012) with experimental data from two strains of *Nannochloropsis* (*N. oculata* and *N. salina*) averaged over each month in the same manner as for the irradiance description. Nutrients in all simulations were supplied, as per Quinn et al. (2012), at 5 × *f*/2 (Guillard and Ryther 1962; Guillard 1975) concentrations (i.e. 61.6 mg N L^−1^ and 5.5 mg P L^−1^). Effects of harvest timing were investigated by considering three different strategies: daily harvesting or every 4th or 7th day (akin to a batch mode).

Despite the comprehensive documentation of the production and irradiance data sets, three of the parameters crucial for model operation (Dil, ChlC_m_ and *μ*_max_; see Fig. 2) were not measured experimentally. To fill this knowledge gap, values of these three inputs were estimated by using the parameter tuning software Powersim Solver v.2 (Isdalstø, Norway). In this approach, the values of input parameters are systematically varied using stochastic methods until a “best-fit” to the data is achieved. Here, these three parameters, as inputs to the model describing full variable C:N:P:Chl stoichiometry, were tuned so that the predicted average volumetric production of bulk biomass over a growth period of 1 year matched that achieved experimentally as closely as possible.

Temperature is an important factor affecting algal growth. In the model of Flynn (2001), temperature is most readily included as a *Q*_10_ multiplier on the maximum growth rate (*μ*_m_). The value of that multiplier is by convention expected to be around 2 (i.e. an increase in temperature by 10 °C would double *μ*_m_). Tuning of the model against the data of Quinn et al. (2012) gave an estimated value for *μ*_m_ = 1.04 day^−1^ over winter and *μ*_m_ = 1.5 day^−1^ over summer which would reflect changes in the operating temperature for the system. The magnitude of these values is consistent with those measured for other strains of *Nannochloropsis* (Griffiths et al. 2011). The difference between the winter and summer estimates of *μ*_m_ equate to a *Q*_10_ of 2.5 assuming a 5 °C difference in temperature between winter and summer operation, which agrees with the data from this particular growth system (Quinn et al. 2011). There are insufficient data from which to develop a more complete growth-temperature model in this instance, but these values for *μ*_m_ are consistent both with what we do know about the production system and also about growth responses to changes in temperature.

Tuned values for the dilution rate varied depending upon the harvesting frequency chosen (every 1, 4 or 7 days) and *μ*_m_ (Dil must be moderated to prevent slow growing cells from being flushed out of the system). The maximum value of Chl:C (ChlC_m_) was estimated to be 0.033 g Chl g^−1^ C, which again is a reasonable value (Flynn 2003a). Along with these estimates, the comparisons made between the model predictions for production against the legacy data also required informed assumptions that carbon biomass accounts for 50 % of the bulk cellular mass and a fatty acid (as FAME) density of 880 kg m^−3^ (Pratas et al. 2011) with a typical carbon fraction of 75 % (Geider and LaRoche 2002). The model was run within the Powersim Constructor v2.51 (Isdalstø, Norway) platform with an integration step size of 11.25 min, with explicit simulation of day and night irradiance.

## Results

### Average irradiance

**Fig. 3:**
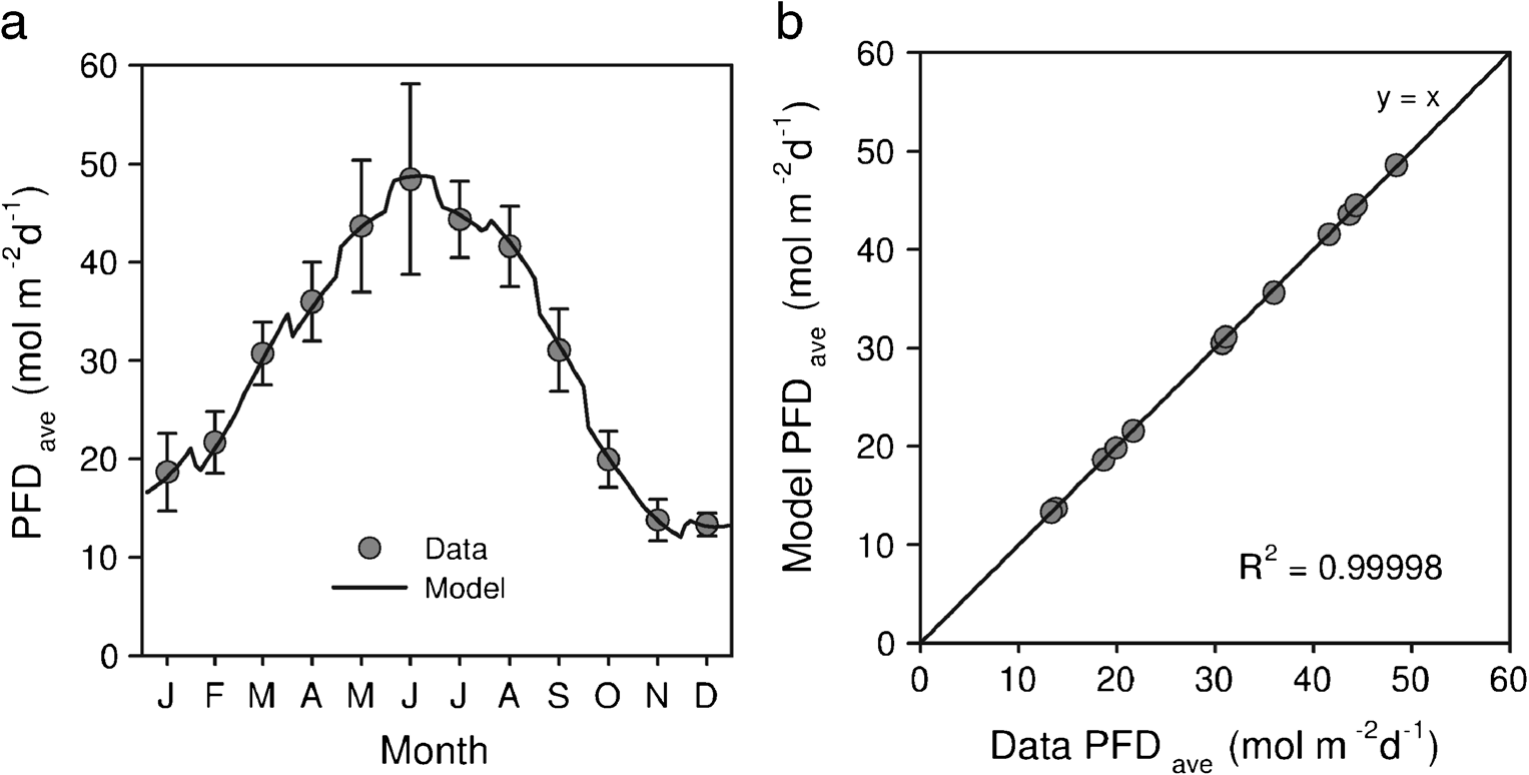
A comparison of the irradiance model to photon flux density (PFD) data recorded over a period of 2 ½ years at the Solix BioSystems research and development facility in Fort Collins, CO, USA (Quinn et al. 2012). **a** Plots the model outputs with average PFD data for each month over the operational period. *Error bars* indicate the spread of observations. The clearness parameter was tuned to optimise the fit; see “Methods”. **b** Compares the model output averaged over each month to the experimental data

Running the modelled solar cycle over a simulation time of 1 year (Fig. 3a), the functional description of average irradiance as photon flux density (PFD in Fig. 3a, averaged over a 3-day period) given by Eq. 3 can be seen to provide an excellent comparison with the experimental data (Quinn et al. 2012). When the average monthly PFD predicted by the model is directly compared to the monthly averaged data (Fig. 3b), the fit is very good with a calculated (zero intercept) *R* ^2^ = 0.999. Hence, the numerically undemanding description given by Eq. 3 appears to be quite adequate for computing the average irradiance over each panel surface.

### Predicting production

**Fig. 4:**
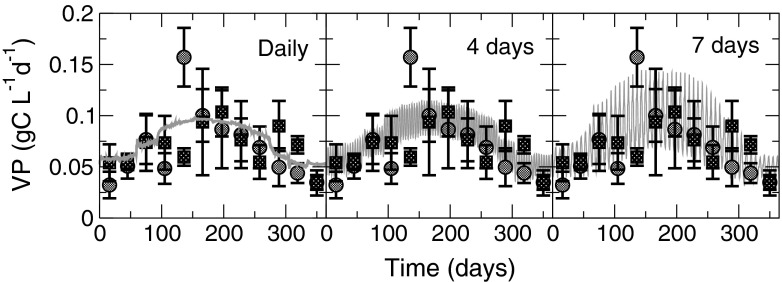
A comparison of modelled volumetric production (VP, *continuous grey line*) to data for *N. oculata* (*circles*) and *N. salina* (*squares*) cultivated at the Solix BioSystems R&D facility in Fort Collins, CO, USA (Quinn et al. 2012). Three cultivation strategies are simulated with harvesting and dilution every 1, 4 and 7 days; the model projection clearly indicates the range of biomass varying with the harvesting periodicity (i.e. small variation with daily harvesting to large variation with weekly harvesting). The experimental data have been adapted from Quinn et al. (2012) by assuming a 50 % C:dw content and then averaged to obtain mean monthly production values for each strain. The *error bars* on the data points indicate the standard deviation in measured production rates

The daily volumetric production (VP) predicted by the model is in good agreement with the experimental data for both *Nannochloropsis* strains (Fig. 4) even without a fully detailed strain-specific parameterisation. Mean daily productivity averaged over the whole year for all three harvesting strategies is VP = 75 mg C L^−1^ day^−1^, within 1 % of the averaged experimental value over the entire data set recorded by Quinn et al. (2012). Peak instantaneous VP in the simulations varies depending upon the harvest frequency chosen, rising from 98 mg C L^−1^ day^−1^ with daily harvesting to 146 mg C L^−1^ day^−1^ for weekly harvesting.

**Fig. 5:**
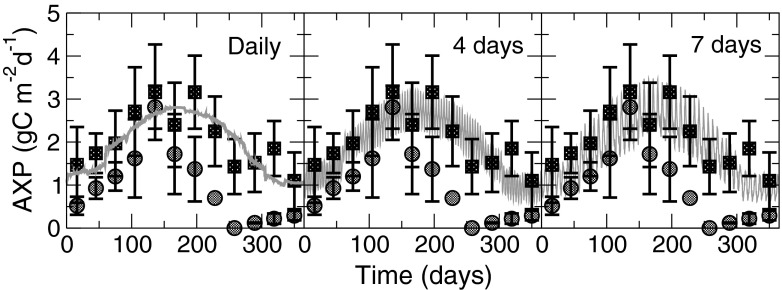
As Fig. 4 but for areal production (AXP) of C-rich biomass (e.g. FAME) components. The experimental data have been adapted from Quinn et al. (2012) by assuming the fatty acid has density of 880 kg m^−3^ and contains a carbon fraction of 75 %. The experimental data are averaged to obtain mean monthly production values for each strain, and the *error bars* on each point indicate the standard deviation in measured production rates. See also the legend for Fig. 4

Concurrent calculations for the areal production of biofuel feedstock components (AXP) produces predictions which again provide a favourable comparison to the experimental data (Fig. 5). Average areal production is 1.96, 1.9 and 1.8 g C m^−2^ d^−1^ for harvest frequencies of 1, 4 and 7 days, respectively. This compares to a calculated mean experimental production of 1.91 g C m^−2^ d^−1^ applying the assumptions made in the Methods section to the data from Quinn et al. (2012). In a similar way to VP (Fig. 4), modelled values for peak instantaneous AXP increase from 2.8 g C m^−2^ day^−1^ with daily harvesting to 3.5 g C m^−2^ day^−1^ for weekly harvesting.

### Comparisons between biological descriptions

**Fig. 6:**
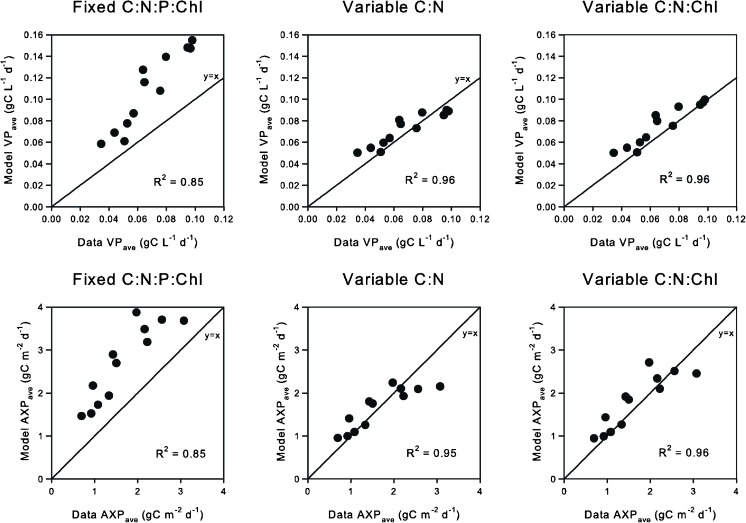
A direct comparison of three biological model descriptions (see “Methods”) to VP and AXP data from Quinn et al. (2012). Simulation data are averaged to obtain mean monthly values. Models assuming fixed stoichiometry fail to give such a good fit as do those featuring some degree of varying stoichiometry (either variable C:N or C:N:Chl)

The adequacy of the three less sophisticated biological model descriptions was tested by comparing their predictions of average daily VP and AXP over each month with the experimental averages over the whole data set. When the model outputs are plotted directly against the data (Fig. 6), two of the three models (those with some form of varying cellular elemental stoichiometry) correspond well. The remaining model (where stoichiometry is fixed) consistently over-estimates production, in total by >40 %. The models with varying C:N and C:N:Chl fit equally well when predicting VP, but the slightly higher *R* ^2^ value for fitting to AXP suggests that the latter model has predicted biofuel production marginally more accurately on this particular occasion.

## Discussion

The data sets of Quinn et al. (2012) are an invaluable resource as they record long-term production of bulk biomass and, uniquely, biofuel feedstocks beyond the laboratory scale. As such, they provide a good test of any proposed model description of long-term production of algal production at a commercial scale. Even so, a full, robust strain-specific, tuning of the model could not be performed in this instance as the stoichiometric data required to enable this is not available. In consequence, we used default estimates based upon our previous deployment of the model (see Appendix B), and made simplified assumptions regarding the carbon content and fatty acid (FAME) density. Through such steps, we obtained a predicted average daily biomass productivity within a 1 % margin of error and biofuel productivities within 6 % against the reported experimental averages (Quinn et al. 2012). The effectiveness of the photoacclimative model with varying C:N:P:Chl stoichiometry to simultaneously reproduce the irradiance data and accurately predict biomass and biofuel feedstock production rates gives confidence that the overall construct is sound. The results validate the modelling approach, especially with regards to testing the description of the production of carbon-rich biomass components (such as fatty acids) for biofuel feedstocks (Flynn et al. 2013) in an industrial biotechnology setting. This validation also gives support to the commentary given by Kenny and Flynn (2015) for the potential for industrial-scale solar-powered production of algal biomass and biofuels in open ponds using the same physiological model.

A balance needs to be struck between complexity and adequacy for both the sub-models describing the optics and the algal productivity. Very often in the arena of applied phycology (and indeed in oceanography), more computational effort is expended on the abiotic description. This raises the question (as developed in the “Introduction”) as to whether the algal sub-model is adequate for the task at hand. The simplest algal production models assume fixed efficiencies for photosynthesis, while the simplest recognisable description of algal physiology assumes fixed stoichiometry. Our results (Fig. 6) show that a fixed stoichiometric model operates at significant variance from reality. However, our results (Fig. 6) also suggest that an absolute necessity for the fully featured photoacclimative model is not proven in this instance. Provided the relationship between nutrient uptake kinetics, cell nutrient quota and growth is adequately defined (Flynn 2008a, b), a less comprehensive (but still relatively complex) model incorporating varying C:N, as a minimum requirement, should suffice as a first approximation. Indeed, the pigment level can be related to cellular C:N without recourse to a full dynamic description of Chl:C (Flynn 2003a).

The minimum requirement for modelling production of biofuel, or other high-C feedstock components such as fatty acids, includes variable C:N stoichiometry (Flynn et al. 2013). If a model with fixed stoichiometry is used, then AXP could only be estimated with a priori knowledge of the average fatty acid content (in this case, approximately 33 % of dry weight matter by reference to the data (Quinn et al. 2012) and by imposing it as a predefined constant proportion of biomass. As this model overestimated both VP and AXP by some margin (Fig. 6), such an approach would seem unjustified. This serves to emphasise the value of a mechanistic approach to modelling algal physiology and production, rather than relying on empirical models. Here the bulk of the mechanistic algal model was configured with generic parameters; although a better fit could no doubt have been achieved had appropriate data been available, mechanistic descriptions provide clear advantages over crude descriptions that need to be configured independently for each site and operational situation.

When making projections for production potential, it is important to consider the contrast between instantaneous and average productivities. For example, Fig. 4 shows the peak instantaneous biomass VP to be achieved with weekly harvest intervals (146 mg C L^−1^ day^−1^ cf. 98 mg C L^−1^ day^−1^ for daily harvest) and yet (see “Results”) that strategy ultimately produced no more biomass over the course of 1 year than did a daily harvesting regime, only greater variability in production rates. Mean daily production (both VP and AXP) averaged over the whole year was little over half the peak value using a weekly harvest, whereas daily harvesting resulted in a mean production rate around 70–80 % of the peak value. Thus, care must be taken not to place too much emphasis on isolated (possibly unrepeatable) yields and attempting to base long-term production projections on short-term results. We would suggest that the deployment of a comprehensive modelling approach (such as that we deploy here) provides a much more secure route for computer-aided modelling and design deliberations over how best to build and operate large-scale commercial microalgal enterprises than do models which attempt to determine production rates using a fixed photosynthetic efficiency (Weyer et al. 2010) or only considering the dynamics of external factors (Béchet et al. 2013).

The two-line description contained in Eqs. 1 and 3 of the average irradiance incident to a photobioreactor with a regular geometry is very simple to incorporate into any model of production utilising a flat panel PBR, and it is computationally undemanding. While the physics of the problem is of interest to many, our results suggest that much of the detail captured in more complicated irradiance models than used here is likely superfluous in considerations of simulating microalgal productivity in industrial-scale systems. While such complexity may be more justified when exploring innovative lighting solutions for specific applications at the laboratory scale (Cornet and Dussap 2009; Takache et al. 2012), it can be seen from our work to be more (or at least equally) worthwhile to ensure the biological description is functional before going too deeply into the physical description of the environment. That is particularly so given the intimate feedback interactions between light availability, biomass growth and light attenuation by the accumulating (pigmented and photoacclimating) biomass.

Given that the algal model thus needs to describe changes in Chl content (i.e. Chl:C) in response to light and nutrient limitations, and that the latter then requires variable N:C (and/or P:C), then this would indicate that the most logical algal model configuration may indeed need to report variable C:N:Chl (or C:N:P:Chl). Such a model can also readily provide an estimate of fatty acid content (from change of the N:C from the optimal value seen in N-replete cells). While the present discussion concerns descriptions of the light-growth relationship, it can be argued that if knowledge of the impact of temperature is not cross related to a knowledge of nutrient and photo-physiology, then the effort in constructing a temperature-growth model is similarly wasted. To take such descriptions beyond a site- or strain-specific empirical model, a mechanistic model describing nutrient and photo-physiology is required. The utility and effectiveness of the variable stoichiometric, photoacclimative model we used here is proven and well documented (see Appendix B) and operates with a lower step time than do complex models of optics.

In conclusion, we suggest that any modelling of microalgal growth in an industrial setting would be best to include a dynamic variable stoichiometric (e.g. C:N:P:Chl) description of algal physiology. As growth and stoichiometry affect productivity at a more fundamental level than at just the light field, there appears little logic in extending the description of the light field in models that do not provide a dynamic description of the growth and stoichiometry of the algae.

## Electronic supplementary material

Below is the link to the electronic supplementary material.

ESM 1 (DOC 155 kb)

ESM 2 (XLS 57 kb)
