# Supplementary material for: Coupling a simple irradiance description to a mechanistic growth model to predict algal production in industrial-scale solar-powered photobioreactors
Source: J Appl Phycol. 2016 Jun 21;28(6):3203–12. doi: 10.1007/s10811-016-0892-6 (PMC5155024; doi:10.1007/s10811-016-0892-6)
Supplement: Supplementary file 1 — (DOC 155 kb) [file 10811_2016_892_MOESM1_ESM.doc]

**Coupling a simple irradiance description to a mechanistic growth model to predict algal production in industrial-scale solar-powered photobioreactors**

**Journal of Applied Phycology**

Philip Kenny & Kevin J. Flynn*

Swansea University, *corresponding author email: k.j.flynn@swansea.ac.uk

**Appendix A: Equations Describing Varying C:N and C:N:Chl Stoichiometry**

The growth model is based upon a description of cell quota dynamics. Under nutrient-limited conditions, growth depends upon the availability of internal nutrients for the cell to utilise, along with available light and the size of the photosynthetic apparatus (as defined by *Chl:C*).

The dynamics of the N:C quota are described thus:


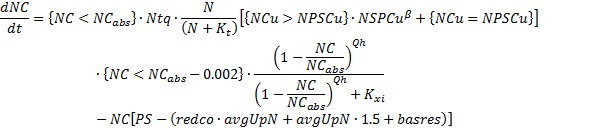


(A.1)

with photosynthesis (PS) as described according to Eqn. 4 in the main text, avgUpN as the average (over n days) N-source uptake, and other parameters as outlined below. The Boolean conditions within { } regulate non-limiting nutrient uptake depending upon the C-specific growth rate NCu and are governed by the outcome of the following:


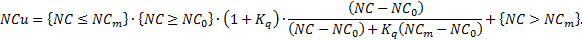


(A.2)

The basal respiration rate is given by:


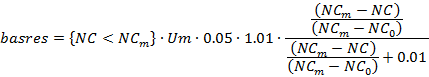


(A.3)

N-source uptake is described by:


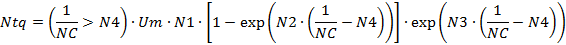

 (A.4)

and


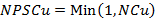


(A.5)

The dynamics of photoacclimation (i.e. changes in the Chl:C quota with light and nutrient-status of the cells) are described as so:


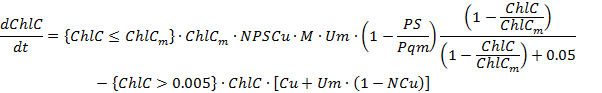


(A.6)

where


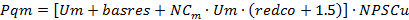


(A.7)

and


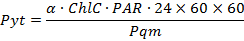

(A.8)

See also Flynn (2001, 2003a, 2003b, 2008a) for further details and how to extend these descriptions to include varying N:C:P:Chl. A list of applications of this model type is given in Appendix B. Table A.1 list parameter values, while Table A.2 summarises different algal model configurations.

**Table A.1** Parameter values for the algal model

| **Name** | **Description** | **Value** | **Units** |
| --- | --- | --- | --- |
| NC_abs_ | absolute maximum algal N:C | 0.25 | gN/gC |
| NC_m_ | maximum algal N:C affecting growth rate | 0.15 | gN/gC |
| NC_0_ | minimum algal N:C | 0.05 | gN/gC |
| K_t_ | half saturation for DIN usage | 14 | mgN/m3 |
| β | power for control of non-limiting nutrient | 0.05 | - |
| Qh | controller of non-limiting nutrient | 2 | - |
| K_xi_ | controller of non-limiting nutrient | 0.001 | - |
| redco | C respired to support nitrate reduction through to intracellular ammonium | 1.71 | gN/gC |
| K_q_ | normalised N:C quota control of algal growth | 10 | - |
| Um | Maximum growth rate | 1.04/1.5 | d^-1^ |
| N1 | Constant defining nitrate transport related to N:C | -0.29 | - |
| N2 | Constant defining nitrate transport related to N:C | 1.7644 | - |
| N3 | Constant defining nitrate transport related to N:C | -1.9282 | - |
| N4 | CN at no nitrate uptake | 5.47 | gC/gN |
| ChlC_m_ | maximum Chl:C | 0.033 | gChl/gC |
| M | scalar for controlling photoacclimation | 2 | - |
| α | Chl-specific initial slope to PI curve, giving gC fixed per gChl.a per photon | 7x10^-6^ | (m^2^g^-1^ chl.a)(gC umol^-1^ photon) |

**Table A.2** Summary of alternate model configurations. Where the algal composition was not fixed, the ratios of N,P,Chl are given with respect to C-biomass, varying dynamically within the ranges indicated according to the mechanistic basis of the model description (developed from Flynn 2001). All ratios are as mass. Only models with variable N:C can describe dynamic changes in C-content (storage carbohydrate and/or fatty acids) such as those identifiable as “biofuels”. Only models with variable Chl:C can describe dynamic photoacclimation, responding to self-shading and N-limitation by altering cellular pigmentation. Only models with variable P:C can describe growth varying with different nutrient N:P ratios.

| Model | Fixed stoichiometry | Variable stoichiometry | N:C | P:C | Chl:C |
| --- | --- | --- | --- | --- | --- |
| Full |  | C:N:P:Chl | 0.05-0.25 | 0.005-0.04 | ≤0.033 |
| Varying C:N:Chl | C:P | C:N:Chl | 0.05-0.25 | 0.01 | ≤0.033 |
| Varying C:N | C:P:Chl | C:N | 0.05-0.25 | 0.01 | 0.033 |
| Redfield | C:N:P:Chl |  | 0.16 | 0.01 | 0.033 |
